# SMoLR: visualization and analysis of single-molecule localization microscopy data in R

**DOI:** 10.1186/s12859-018-2578-3

**Published:** 2019-01-15

**Authors:** Maarten W. Paul, H. Martijn de Gruiter, Zhanmin Lin, Willy M. Baarends, Wiggert A. van Cappellen, Adriaan B. Houtsmuller, Johan A. Slotman

**Affiliations:** 1 Erasmus Optical Imaging Centre, Erasmus MC, Wytemaweg 80, 3015 CN Rotterdam, The Netherlands; 2 Department of Pathology, Erasmus MC, Wytemaweg 80, 3015 CN Rotterdam, The Netherlands; 3 Department of Neuroscience, Erasmus MC, Wytemaweg 80, 3015 CN Rotterdam, The Netherlands; 4 Department of Developmental Biology, Erasmus MC, Wytemaweg 80, 3015 CN Rotterdam, The Netherlands

**Keywords:** Single-molecule localization, Microscopy, Image quantification, Image analysis, Super-resolution, R

## Abstract

**Background:**

Single-molecule localization microscopy is a super-resolution microscopy technique that allows for nanoscale determination of the localization and organization of proteins in biological samples. For biological interpretation of the data it is essential to extract quantitative information from the super-resolution data sets. Due to the complexity and size of these data sets flexible and user-friendly software is required.

**Results:**

We developed SMoLR (Single Molecule Localization in R): a flexible framework that enables exploration and analysis of single-molecule localization data within the R programming environment. SMoLR is a package aimed at extracting, visualizing and analyzing quantitative information from localization data obtained by single-molecule microscopy. SMoLR is a platform not only to visualize nanoscale subcellular structures but additionally provides means to obtain statistical information about the distribution and localization of molecules within them. This can be done for individual images or SMoLR can be used to analyze a large set of super-resolution images at once. Additionally, we describe a method using SMoLR for image feature-based particle averaging, resulting in identification of common features among nanoscale structures.

**Conclusions:**

Embedded in the extensive R programming environment, SMoLR allows scientists to study the nanoscale organization of biomolecules in cells by extracting and visualizing quantitative information and hence provides insight in a wide-variety of different biological processes at the single-molecule level.

**Electronic supplementary material:**

The online version of this article (10.1186/s12859-018-2578-3) contains supplementary material, which is available to authorized users.

## Background

The revolutionary advancements in super-resolution microscopy techniques make it possible to study subcellular structures at nanoscale, using fluorescence microscopy. Single-molecule localization microscopy (SMLM) provides the highest spatial resolution that can be achieved with light microscopy today, with a lateral resolution between 10 and 20 nm [1, 2]. SMLM relies on detecting single fluorescent emitters, by separating spatially overlapping signals in time. By detecting and determining the position of individual fluorescent molecules, in densely labelled biological samples, with high precision, images can be reconstructed with a resolution an order of magnitude below the diffraction limit of the light microscope.

In many biological samples a multitude of macromolecular assemblies and protein complexes within one cell can be observed, such as DNA double strand break (DSB) foci [3, 4], nuclear pores [5], focal adhesions [6], virus particles [7] or neuronal spines [8]. Super-resolution microscopy is well suited to study those assemblies, since the increased resolution permits to investigate, at the single-molecule level, the internal composition and protein distribution of these nanoscale assemblies, which have typical diameters ranging from 100 nm up to 2 μm.

In contrast to regular microscopy data which consists of intensity values in a digital image format, SMLM data typically consists of Cartesian coordinates with corresponding localization precision. Therefore, regular image analysis tools do not directly apply to SMLM data. Numerous software packages for detection and localization of single-molecules from single-molecule localization data are available (reviewed and benchmarked in [9]), that allow reliable image reconstruction for SMLM. Additionally tools have been developed which allow more in-depth (3D) visualization of the localization data (PALMsiever [10], ViSP [11], PYME [12]), clustering (SR-Tesseler [13], 3DClusterVisu [14]) and extraction of quantitative information (SharpViSu [15], LAMA [16] and Grafeo [17]) (Table 1).

**Table 1:** Comparison of different software packages for visualization and analysis of Single Molecule Localization data

	Programming environment	Visualization	Clustering/ segmentation	Quantification	GUI	Batch mode/Scriptable	Reference
VisP	C++	+	–	–	+	–	[11]
PALMsiever	Matlab	+	–	–	+	+	[10]
SR-Tesseler	C++	+	Voronoi	+	+	–	[13]
PYME	Python	+	–	–	+	+	[12]
SharpViSu	Matlab	+	Ripley/Voronoi	+	+	–	[15]
LAMA	Python	–	Ripley/DBSCAN	+	+	–	[16]
3DClusterViSu	Matlab/Python	+	3D Voronoi	+	+	+	[14]
Grafeo	Matlab	+	Ripley/Voronoi	+	+	+	[17]
SMoLR	R	+	KDE/DBSCAN	+	+	+	this paper

Here, we present a versatile software package named SMoLR (Single Molecule Localization in R), that enables researchers to analyze large sets of single-molecule localization data in a quantitative way. The pointillist nature of the data gives possibilities for alternative types of analysis, for which the resourceful R programming language can be of great value [18]. With SMoLR we complement existing software, with a software package for analyzing larger data sets with localization data at once in the free open-source R environment.

## Implementation

**Fig. 1:**
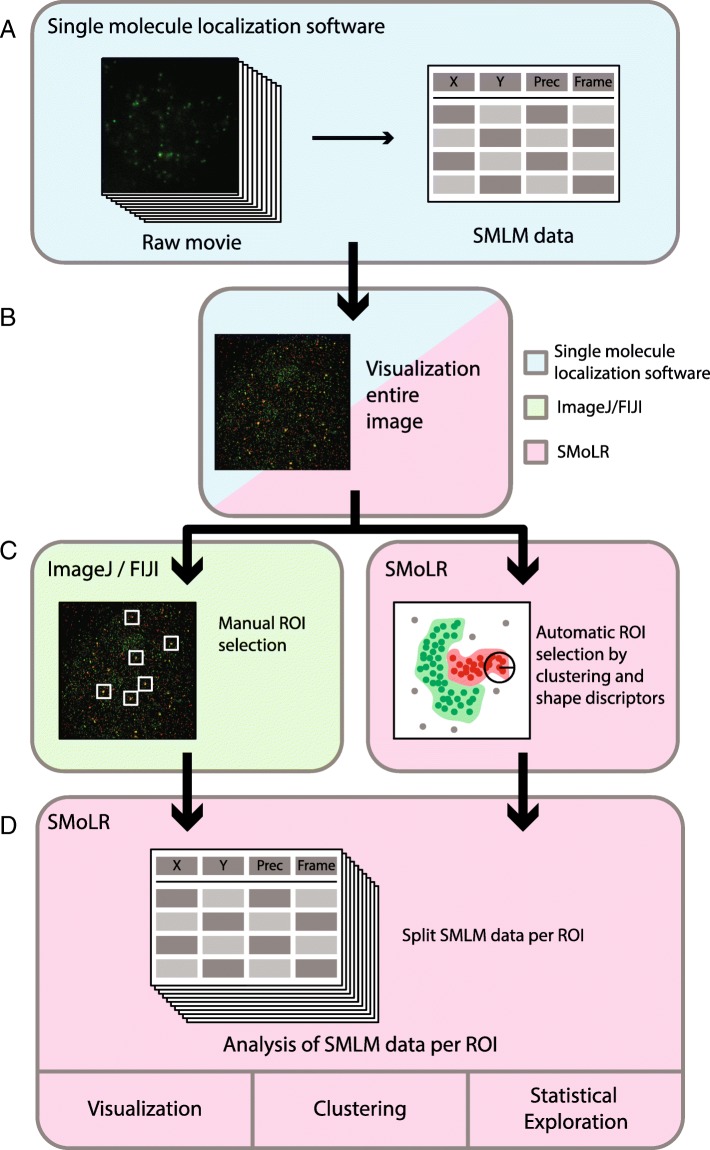
Workflow for analysis of SMLM data with SMoLR. Workflow across external single-molecule localization software (blue), ImageJ/FIJI (green) and SMoLR (pink). (**a**) SMLM data is extracted from microscopy images, and represented in table format with as minimum information x and y coordinates, and localization precision. (**b**) The extracted data is analyzed with SMoLR or other visualization programs as an entire image. (**c**) Using either ImageJ/FIJI or SMoLR regions of interest are determined and selected, either manually or automatically using selection criteria. (**d**) The localization data is split by SMoLR into a list containing data of each ROI, these individual ROIs can be analyzed in more detail at once. Resulting parameters can easily be statistically explored using R

SMLM data consist of Cartesian coordinates of molecules and their respective precision along with all possible extra information that is desired in a specific experiment (i.e. time or frame of detection, channel, estimated number of photons detected etc.). The localization data together with these additional parameters can be imported into SMoLR in different formats obtained by different single-molecule localization software: ThunderSTORM [19], Zeiss ZEN software, SOSplugin [20] or plain text (Fig. 1). SMoLR is versatile and can be used in different ways, where one specifically useful way is to define Regions of Interest (ROIs) from the super-resolution images to analyze the organization of proteins in subcellular structures. Subsequently applying a single analysis to each ROI will result in quantitative information describing the distribution of proteins in a large number of structures.

### Workflow

ROIs can be either manually or automatically selected in image analysis software such as ImageJ [21], the localization data of these ROIs can be imported in SMoLR (Fig. 1). Alternatively, ROIs can also be automatically selected using localization clustering functions in SMoLR. The localization data within the different ROIs is selected and stored in a list with localization data from the different ROIs. These objects can subsequently be analyzed by SMoLR at once, using single commands. To visually inspect the ROI data, we provide an interactive application which shows the ROIs in the full super-resolution image together with several statistical parameters (Additional file 1: Figure S1).

### Visualization

**Fig. 2:**
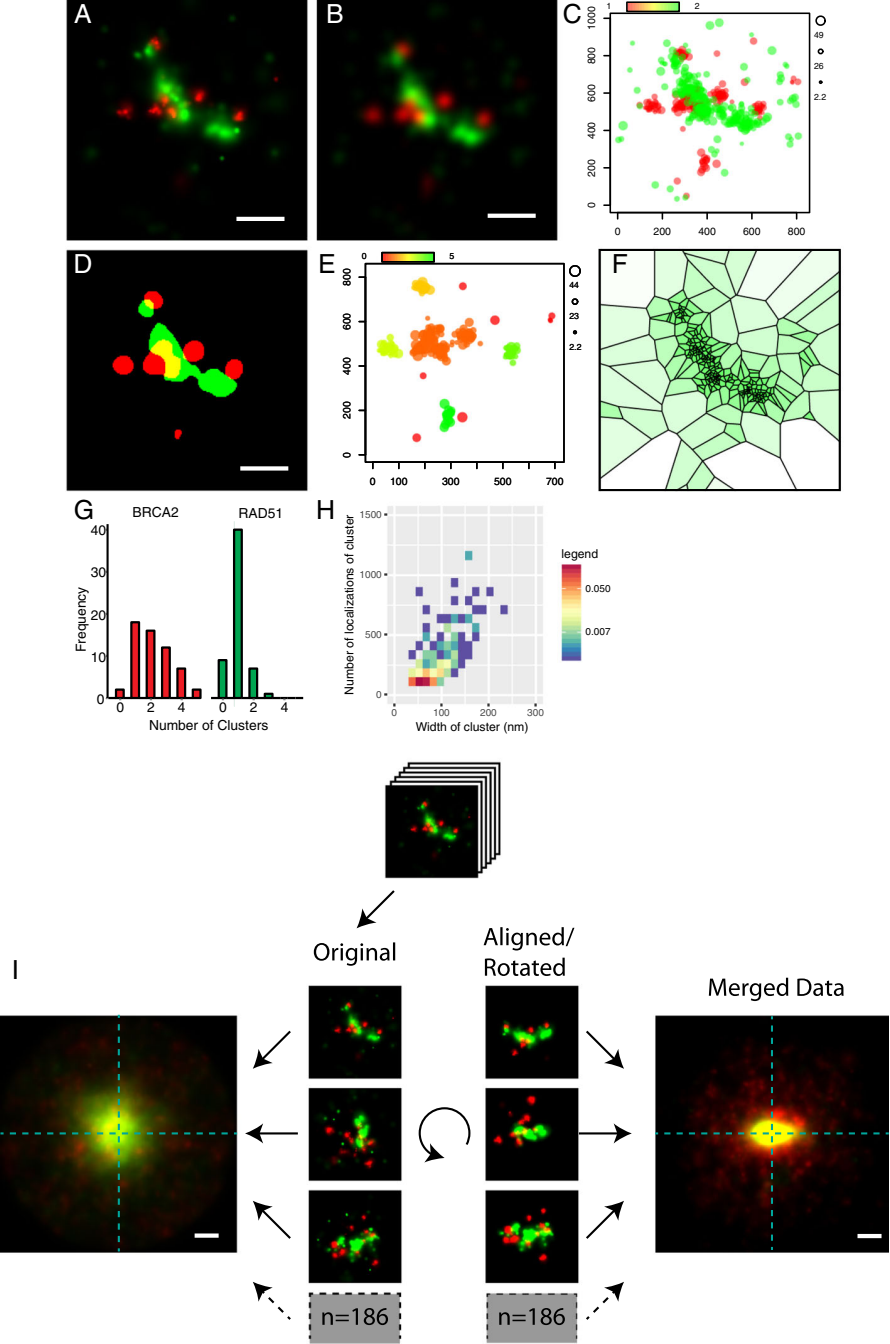
Analysis of DSB foci with SMoLR Human (U2Os) cells were indirectly immunostained for RAD51 (Atto488, green) and BRCA2 (Alexa647, red) and imaged by dual color dSTORM. Visualization (**a**-**c**), clustering (**d**-**f**) and statistical exploration (**g**-**h**) as featured in SMoLR is shown. (**a**) Single DSB foci plotted as Gaussian distributions, (**b**) kernel density estimation and (**c**) as an extended scatter plot where the size of the points represents the localization precision. Three clustering algorithms: (**d**) KDE, (**e**) DBSCAN and (**f**) Voronoi tessellation. Clusters are shown in separate colors for KDE and DBSCAN. Voronoi tessellation is depicted with a color intensity that correlates with area of the tiles, hence the local density of localizations. Graphical representations of cluster information: (**g**) Histogram with the number of clusters per DSB focus for the two proteins, (**h**) 2D histogram of cluster Size (FWHM) versus number of localizations of the BRCA2 foci. (**i**) Template free particle averaging of multiple (*n* = 186) DSB foci; the center and orientation of the RAD51 signal was determined and used to align and rotate the foci. Additionally the foci were oriented in such a way that their highest intensity was at the left side of the merged image. For reference, the crosshair indicated the center of rotation. Scale bars are 200 nm

SMLM data can be visualized in many ways. The most frequently used method is to plot Gaussian distributions for all localizations with standard deviations corresponding to the localization precision (Fig. 2a) [22]. However, with this method intensity values do not directly depend on the density of localizations, but also depend on localization precision. As an alternative approach we implemented a 2D-Kernel density estimation (KDE) method, in which the density of detections per area is normalized to the total number of localizations in the images (Fig. 2b). Therefore, this method is quantitative, making thresholding of the data at a given density of localizations per pixel possible. A third visualization method implemented in SMoLR is an adapted scatter plot that depicts the Cartesian coordinates and can add additional data using the size and color of the plotted points (Fig. 2c). This type of visualization can be used to easily assess the quality of the data and detect potential artefacts such as drift during image acquisition or incorrect grouping. Additionally, we provide a function that formats the single-molecule data in such a way that it can be used in the Spatial Point Pattern Analysis R package spatstat [23]. This opens up the possibility to also include spatstats’ wide range of visualization and clustering options in the analysis.

### Clustering

Clustering of SMLM data is comparable to object segmentation in conventional image analysis. Similar to the analysis of objects from segmented images, features can be extracted from the clustered objects to describe the shape and spatial organization within the object. For SMLM data several different approaches for clustering have been proposed in literature, where some of the algorithms are useful to give a global description in the amount of observed clustering, such as Ripley’s K and its derivates, or the recently nonparametric descriptor, J0(r) for clustering density [24]. As previously mentioned, from within SMoLR, the R-package spatstat offers several of these clustering and correlation methods (Ripley-K function, linearized L-function and pair-correlation functions). However, in general, identification of individual clusters is preferred because this allows to analyze the size, shape and spatial distribution of the clusters. In SMoLR, multiple clustering algorithms are available. First, a clustering method based on the binary KDE image can be used to quantify the number of clusters in an image or region of interest (Fig. 2d). We incorporated functions from the EBImage package to calculate image features, such as shape and size, from single clusters [25]. These features together with descriptive statistics (number of localizations, mean position, mean precision, etc.) can be used to categorize individual clusters. Second, the Density Based Clustering Algorithm with Noise (DBSCAN) algorithm is integrated in SMoLR (Fig. 2e) [26, 27]. This frequently used algorithm allows clustering of data based on localization data only. From the defined clusters with localizations, statistics can be calculated such as the cluster area, convex hull and elongation. The earlier mentioned interactive application (Additional file 1: Fig. S1) at this point also allows to manually assess the features (obtained with KDE or DBSCAN clustering) within a data set. Additionally, all parameters can be used for exploration of the data set either manually or using multivariate analysis or machine learning algorithms. Although DBSCAN is able to define clusters and deal with noise, in literature alternative clustering algorithms have been proposed that work better for certain biological samples. Examples are Voronoi tessellation, Bayesian cluster identification and the use of a Gaussian-mixture model [13, 28–30]. A comparison of our KDE and DBSCAN implementations with clustering algorithms by Voronoi tessellation [13, 17] and Bayesian statistics [29] can be found in Additional file 2: Figure S2.

### Particle averaging

Merging the localizations from a large number of individual SMLM images of single biological structures such as the nuclear pore complex, synaptonemal complex or viral particles proved to be a powerful tool to reconstruct ultrastructure [5, 31–33]. However, template free particle averaging is a computationally demanding procedure or requires expensive software [33]. Particle averaging also assumes that individual structures represent identical or at least highly similar structures. However, for some biological structures there might be quite some variation in the organization of the individual structures, although they can have certain features in common. We therefore implemented an alignment algorithm, as will be described below, based on extracted features from the individual images, which can be very informative to observe common features from the imaged structures.

Alignment of individual structures can be achieved using features that can be extracted with the SMoLR package (using pixel- or localization-based features). For example, the center of mass of clusters can be used to center the structures. In some cases, the clusters may have specific shapes that enable to rotate and overlay the individual ROIs. For example, elongated structures can be aligned using the major axis of the structure. The presence of multiple clusters within individual ROIs that can be distinguished from each other (for instance on the basis of shape, size or distance to the center of mass), provides another possibility to align structures by rotating the similar clusters towards the same point. The alignments can be averaged or overlaid, and subsequently used to visualize and extract common features from the individual images. This can be used to compare biological structures at different biological conditions or time points. Additionally, these alignments can reveal the relative location of different proteins within the structure, when aligning the structures using one protein as a reference.

The functions in SMoLR are developed based on 2D-localization data. However, 3D data can be visualized in the scatterplot of SMoLR visualizing the z-coordinate using color or size of the plotted points. In principle the DBSCAN algorithm is not limited to 2D data, however 3D clustering is not implemented directly in SMoLR.

## Results

To show the use of SMoLR to analyze single-molecule localization data, we applied the functions of the SMoLR package on a previously published data set with images of proteins involved in DNA double strand break (DSB) repair [4]. Precise determination of spatiotemporal localization and organization of these proteins at the sites of damage and how these relate to specific and general protein functions can help to elucidate the mechanisms by which repair of the DSBs take place. In this example we examined two essential DSB repair proteins, the recombinase RAD51 and the tumor suppressor BRCA2. γ-Irradiated cells were immunostained for RAD51 and BRCA2 and imaged using direct stochastic optical reconstruction microscopy (dSTORM) [4]. Single foci were segmented and visualized using the three visualization techniques available in SMoLR (Fig. 2a-c). Subsequent clustering using KDE, DBSCAN and Voronoi tesselation (spatstat) (Fig. 2d-f) allowed for quantitative analysis of multiple foci including number of clusters per protein, per focus and cluster size versus number of localizations (Fig. 2g-h). These analyses can be extended using e.g. cluster shape, co-localization or relative distance between clusters.

In order to gain insight in the relative distribution of RAD51 and BRCA2 in DSBs we averaged their signal after alignment (centered and rotated) based on the elongated shape of the RAD51 clusters (Fig. 2i). This revealed a distinct pattern of protein distributions during DNA repair (explained in more detail in Sánchez et al., 2017).

## Conclusions

Visualization and quantitative analysis of the localization of multiple proteins, below the diffraction limit, within macromolecular assemblies or small organelles, under different conditions and at multiple time points, provides the possibility to gain insight in the spatiotemporal organization of protein function during biological processes. In many situations, multiple similar structures are present within a cell and the recorded super-resolution image. By combining the presented methods and work flow to extract relevant features from the localization data, together with the powerful statistics available in R, it is possible to explore the variation in structures, determine common features describing the structures while at the same time comparing different conditions or proteins. Using feature-based alignment and rotational analysis these observed structural organizations can be verified, visualized and combined with simulations to get more insight. Altogether, the workflow presented in our SMoLR package allows researchers to delve deeper into their single-molecule localization data, beyond conventional image analysis.

## Availability and requirements

Project name: **SMoLR**

Project home page: https://github.com/ErasmusOIC/SMoLR

Operating system(s): **Platform independent**

Programming language: **R**

Other requirements: **R 3.4.0 or higher **

License: **LGPLv3**

Any restrictions to use by non-academics: **no**.

## Additional files

Additional file 1: **Figure S1.** Interactive application for inspection of SMLM data (A) Shiny application loaded with indicated data is run within the R environment on a local server in a web browser. (B) Feature parameters can be show in a scatter plot or (C) binned in a histogram. (D) Data points inside the scatterplot or bins in the histogram can be manually selected and corresponding clusters are then indicated in the image (green is selected), structures of interested can be enlarged and inspected. (PDF 944 kb)

Additional file 2: **Figure S2.** Comparison of cluster algorithms: Four cluster algorithms were compared KDE and DBSCAN from the SMoLR package and Voronoi and Bayesian clustering from external packages. (A) A test data set containing 6 circular clusters of 50 localizations (1–6) and one cluster of 100 localization consisting of two overlapping clusters (7) (red dots) and 300 uniformly distributed (incorrect) localizations due to noise. (B-C) KDE, DBSCAN, and Bayesian clustering of the test data set using default settings. For Voronoi clustering, the approach as described in Haas et al. was used, using an implementation in R (a threshold of two times the medial tile area of Voronoi tessellation was used to select clustered localizations). Non-clustered localizations are depicted in red, while clustered localizations are indicated as a separate color per cluster (orange to green) and numbered from 1 to 7. Indicated performance parameters are: 1), the number of individual positive clusters detected (fused clusters are counted as one), 2), number of false clusters identified (arrow), 3), the percentage of noise localizations that have been assigned to a cluster and, 4), the percentage of signal localizations that are assigned to a cluster. (PDF 3804 kb)

## Abbreviations

DBSCAN: Density-based spatial clustering of applications with noise
DSB: Double strand break
dSTORM: Direct stochastic optical reconstruction microscopy
KDE: Kernel density estimation
ROI: Region of interest
SMLM: Single-molecule localization microscopy
SMoLR: Single-molecule localization in R
