# Supplementary material for: SMoLR: visualization and analysis of single-molecule localization microscopy data in R
Source: BMC Bioinformatics. 2019 Jan 15;20:30. doi: 10.1186/s12859-018-2578-3 (PMC6334411; doi:10.1186/s12859-018-2578-3)
Supplement: Supplementary file 1 — Figure S1. Interactive application for inspection of SMLM data (A) Shiny application loaded with indicated data is run within the R environment on a local server in a web browser. (B) Feature parameters can be show in a scatter plot or (C) binned in a histogram. (D) Data points inside the scatterplot or bins in the histogram can be manually selected and corresponding clusters are then indicated in the image (green is selected), structures of interested can be enlarged and inspected. (PDF 944 kb) [file 12859_2018_2578_MOESM1_ESM.pdf]

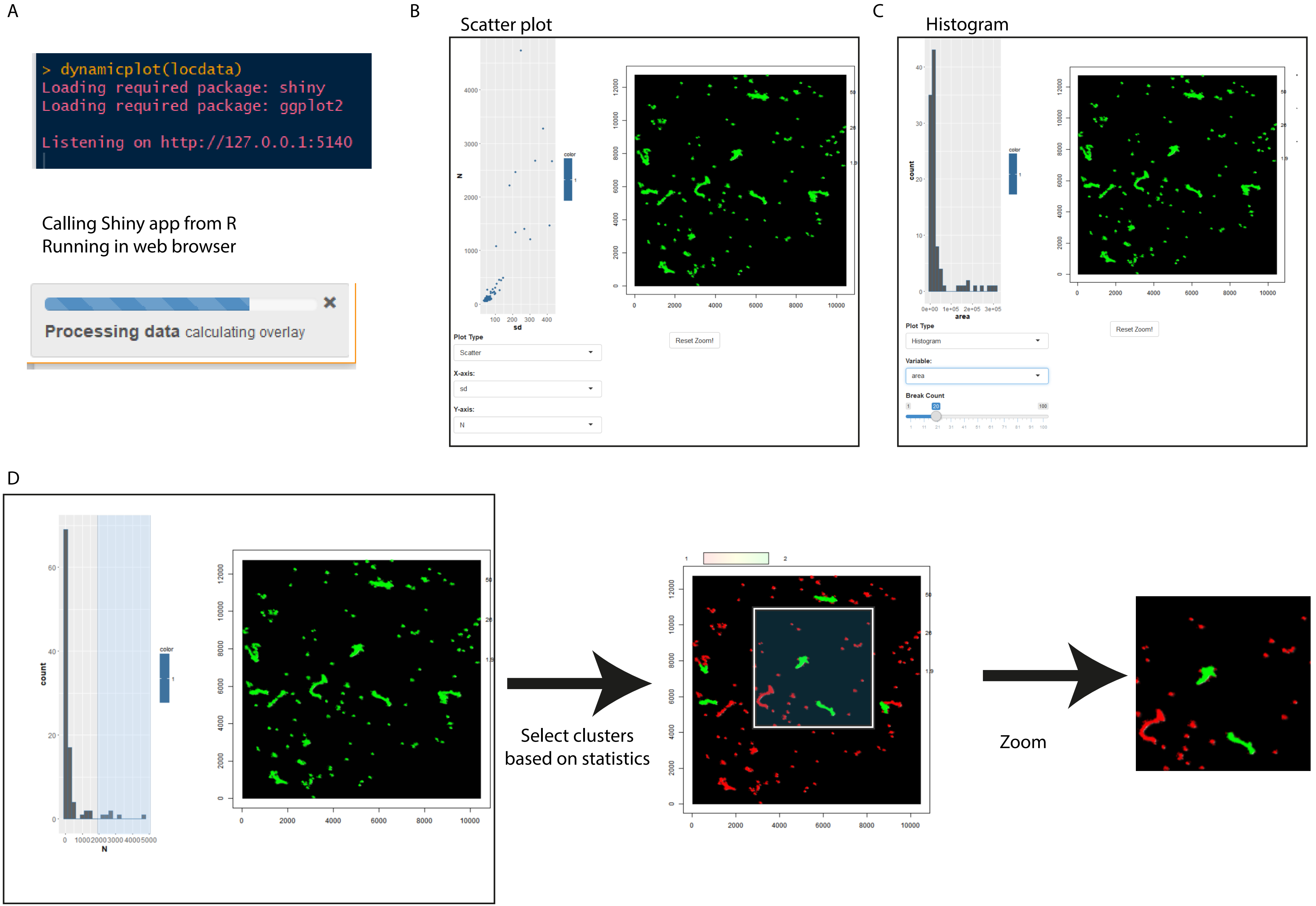
A
> dynamicplot(locdata)
Loading required package: shiny
Loading required package: ggplot2
Listening on http://127.0.0.1:5140
Calling Shiny app from R
Running in web browser
Processing data calculating overlay
B
Scatter plot
Plot Type
Scatter
X-axis:
sd
Y-axis:
N
Reset Zoom!
C
Histogram
count
area
Plot Type
Histogram
Variable:
area
Break Count
Reset Zoom!
D
count
N
Select clusters
based on statistics
Zoom
