# Supplementary material for: SMoLR: visualization and analysis of single-molecule localization microscopy data in R
Source: BMC Bioinformatics. 2019 Jan 15;20:30. doi: 10.1186/s12859-018-2578-3 (PMC6334411; doi:10.1186/s12859-018-2578-3)
Supplement: Supplementary file 2 — Figure S2. Comparison of cluster algorithms: Four cluster algorithms were compared KDE and DBSCAN from the SMoLR package and Voronoi and Bayesian clustering from external packages. (A) A test data set containing 6 circular clusters of 50 localizations (1–6) and one cluster of 100 localization consisting of two overlapping clusters (7) (red dots) and 300 uniformly distributed (incorrect) localizations due to noise. (B-C) KDE, DBSCAN, and Bayesian clustering of the test data set using default settings. For Voronoi clustering, the approach as described in Haas et al. was used, using an implementation in R (a threshold of two times the medial tile area of Voronoi tessellation was used to select clustered localizations). Non-clustered localizations are depicted in red, while clustered localizations are indicated as a separate color per cluster (orange to green) and numbered from 1 to 7. Indicated performance parameters are: 1), the number of individual positive clusters detected (fused clusters are counted as one), 2), number of false clusters identified (arrow), 3), the percentage of noise localizations that have been assigned to a cluster and, 4), the percentage of signal localizations that are assigned to a cluster. (PDF 3804 kb) [file 12859_2018_2578_MOESM2_ESM.pdf]

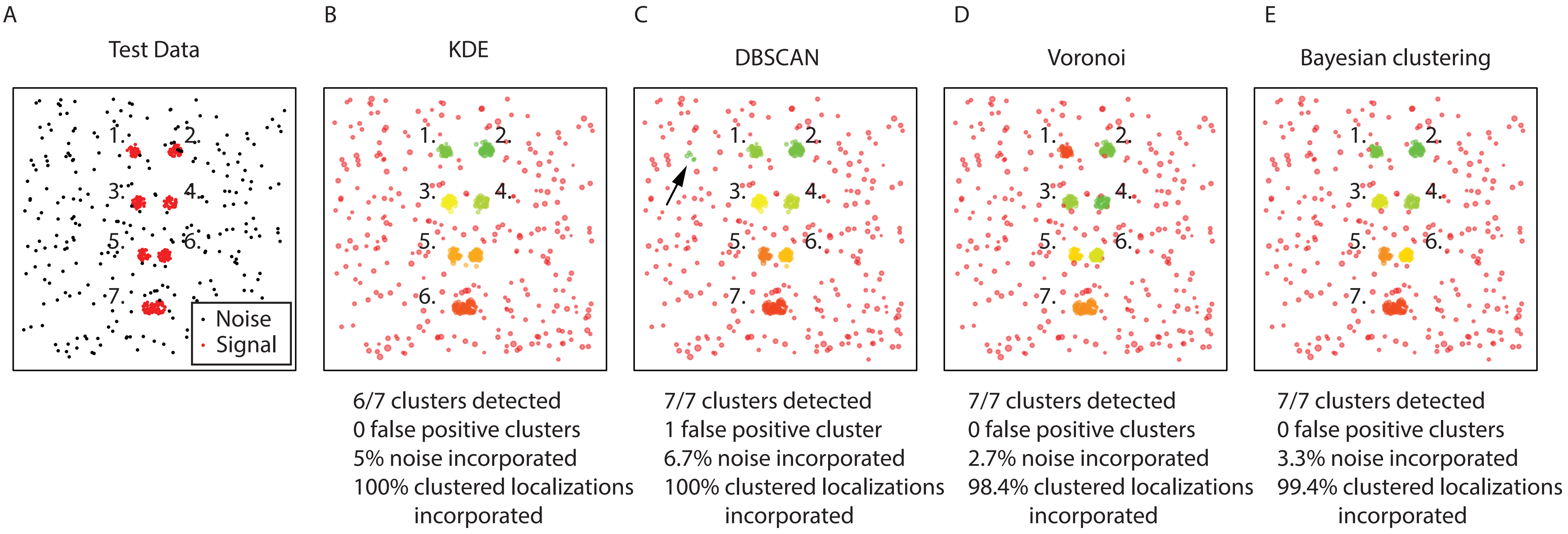
A
Test Data
1.
2.
3.
4.
5.
6.
7.
Noise
Signal
B
KDE
6/7 clusters detected
0 false positive clusters
5% noise incorporated
100% clustered localizations incorporated
C
DBSCAN
7/7 clusters detected
1 false positive cluster
6.7% noise incorporated
100% clustered localizations incorporated
D
Voronoi
7/7 clusters detected
0 false positive clusters
2.7% noise incorporated
98.4% clustered localizations incorporated
E
Bayesian clustering
7/7 clusters detected
0 false positive clusters
3.3% noise incorporated
99.4% clustered localizations incorporated
